# Dissemination and Molecular Characterization of* Staphylococcus aureus* at a Tertiary Referral Hospital in Xiamen City, China

**DOI:** 10.1155/2017/1367179

**Published:** 2017-07-05

**Authors:** Yiwen Yu, Yihui Yao, Qinyun Weng, Jingyi Li, Jianwei Huang, Yiqun Liao, Fu Zhu, Qifeng Zhao, Xu Shen, Jianjun Niu

**Affiliations:** ^1^School of Public Health, Xiamen University, Xiamen, Fujian Province 361102, China; ^2^Zhongshan Hospital, Xiamen University, Xiamen, Fujian Province 361004, China; ^3^Xiamen Center for Disease Control and Prevention, Xiamen, Fujian Province 361021, China; ^4^School of Life Sciences, Xiamen University, Xiamen, Fujian Province 361102, China

## Abstract

*Staphylococcus aureus* is a global epidemic pathogen that causes heavy disease burden. The aim of this study was to determine which globally known* S. aureus* lineages are currently present in a hospital of Xiamen. Therefore, the 426* S. aureus* strains were detected by Melting Curve Analysis (MCA) and genotyped by Pulsed Field Gel Electrophoresis (PFGE) as well as Multicolor Melting Curve Analysis-Based Multilocus Melt Typing (MLMT). In addition, Multilocus Sequence Typing (MLST) was used to identify 108 representative strains. In light of eighteen antibiotics except for Vancomycin (by Broth Dilution Method), we used the Kirby-Bauer disc diffusion method to assess antibiotic susceptibility of 426* S. aureus* strains. Finally, PFGE analysis revealed 14 different patterns with three major patterns (C10, C8, and C11) that accounted for 69.42% of all* S. aureus* strains, and MT-1~MT-5 occupied most part of the strains by MLMT. MLST revealed 25 different STs with the predominant types being ST239, ST59, and ST188. There have been 8 antibiotics that showed more than 50% resistance of all* S. aureus* strains. In summary, we found several of the lineages are predominant in our hospital. And antibiotic resistance is still a severe problem that needs to be controlled in clinic.

## 1. Introduction

*Staphylococcus aureus*, the major pathogen of human infectious diseases which can cause various lesions by secreting a variety of virulence factors, has been becoming a great problem at home and abroad [1]. As we know,* S. aureus* can be isolated from patients with different kinds of diseases, and there are considerable variations in the prevalence of* S. aureus* according to geographic areas. It was proposed by some experts that most* S. aureus* strains belong to a few distinct pandemic lineages which were predominant in hospitals or communities [2].

In recent years, several typing methods have been evaluated for* S. aureus* strains characterization, such as repetitive sequence based PCR (rep-PCR), Pulsed Field Gel Electrophoresis (PFGE), Multilocus Sequence Typing (MLST), Multilocus Variable-Number Tandem Repeats Analysis (MLVA), Staphylococcal Protein A* (spa)*, and Staphylococcal Cassette Chromosome* (SCC-mec)* Typing [3]. Historically, PFGE used to be the “gold standard” of* S. aureus* typing because of convenience and high discriminatory power [4]. At the present time, PFGE is still widely used for short-term and local epidemiology to identify outbreak; however, it is less suitable for long-term and global epidemiology since it was not permitted to compare between different research centers. In addition, PFGE data might change over time and produce weak bands so that it is difficult for us to compare over long time periods or avoid a matter of subjective interpretation [5]. Therefore, another molecular typing method, MLST, served as an auxiliary reference. MLST involves the sequencing of seven housekeeping genes (arcC, aroE, glpF, gmK, pta, tpi, and yqiL) and each unique allelic profile is assigned a sequence type (ST) [6]. Clonal complexes are defined as groups of STs in which every ST shares at least five of seven identical alleles with at least one other ST in the group [7]. In the meantime, as an expensive and time-consuming method, MLST has limitation of usage somewhere. So based on MLST, a new genotyping method called Multicolor Melting Curve Analysis-Based Multilocus Melt Typing (MLMT) were proposed by Li et al. [8] from Engineering Research Center of Molecular Diagnostics, Ministry of Education, State Key Laboratory of Cellular Stress Biology, School of Life Sciences, Xiamen University. The entire MLMT procedure could be finished within 3 hours on a real-time PCR machine and the cost was approximately 50 times less than MLST. The ability of detecting multiple SNPs in one reaction further helps to simplify the operation and increase the throughput. Moreover, the interpretation of *T*_*m*_s into binary codes can be easily automated and the efficiency of detection can be further improved. In this study, all* S. aureus* strains collected in our hospital had been analyzed by PFGE and MLMT from 1 January 2014 to 30 June 2015; also the representatives were identified by MLST. The objectives of the current study were to combine the PFGE data with additional typing methods (MLMT and MLST) and to determine which of the globally known clones were found in our hospital during a one-year-and-a-half study period.

Except for genotyping, the resistance to antibiotics, especially the emergence of multidrug resistant bacteria, has brought great difficulties and challenges to clinical medicine. In 1961, one year after methicillin was applied to clinical therapeutics, methicillin-resistant* Staphylococcus aureus* were isolated from patients and quickly spread throughout the world subsequently. Multiple-drug resistance of MRSA is serious, especially sensitive to glycopeptide antibiotics such as Vancomycin. However, in recent years, it limited the use of glycopeptide antibiotics since the appearance of Vancomycin-intermediate* S. aureus* (VISA) or heterogenous Vancomycin-intermediate* S. aureus* (hVISA). In order to provide the basis for clinical anti-infection treatment, antimicrobial susceptibility should be tested and become an auxiliary method combined with molecular genotyping. The aims of this investigation were to evaluate the molecular and clinical epidemiology of* Staphylococcus aureus* and to explore the transmission between different wards and sickbeds.

## 2. Methods and Materials

This retrospective study was conducted from 1 January 2014 to 30 June 2015 at an 800-bed teaching hospital which provides tertiary care, located in the city of Xiamen, China. With strict personal information protection as conditions, institutional review boards and independent ethics committees of Health and Family Planning Commission of Xiamen approved this retrospective study and the retrospective use of the patient information.

### 2.1. Bacterial Strains

 A total of 458 consecutive, nonduplicate* Staphylococcus aureus* strains were collected including both adults and children and detected by Melting Curve Analysis (MCA) which is based on RT-q-PCR, if the resulting signal peak at 79°C means that the strains carry* FemA* gene* (Staphylococcus aureus)* and if the rising signal peak at 64°C showed that the strains carry* mecA* gene, methicillin-resistant* Staphylococcus aureus* (MRSA). But 24 strains were unavailable because of failure to obtain complete and accurate clinical data; also another 8* S. aureus* strains were excluded depending on 5 of poor quality, 2 of collectors' mistakes, and 1 of loss with efficiency of 93.01%. Thus, there were 426* S. aureus* strains eligible for enrollment, and all of them were identified by standard biochemical test in accordance with Clinical and Laboratory Standards Institute (CLSI) [9]. We isolated the strains from patients with infections as well as from colonized individuals (blood, ascites, bile, secreta, fester, sputamentum, pleural effusion, synovial fluid, drainage liquid, throat swab, and midstream urine, Table 1).

### 2.2. PFGE Typing

The clonal relationships of all 426* S. aureus* strains were assessed by PFGE using* S-maI* as previously described. The PFGE types were defined according to the criteria of Tenover et al. [10]. The dice coefficient was used with 1.25% optimization and 1% tolerance to calculate similarities between PFGE patterns. The strains with >75% similarity were clustered in different patterns. The results were also analyzed using BioNumerics version 6.6.4 statistical software, and dendrograms were generated according to a simple matching coefficient and the unweighted pair group method with the arithmetic mean (UPGMA) algorithm.

### 2.3. MLMT

The dual-labeled, self-quenched probes alone are nonfluorescent or weakly fluorescent but become fluorescent when hybridizing with the reversely complementary single-stranded DNA. After asymmetric PCR, the produced excess single-stranded amplicons are targets for the dual-labeled, self-quenched probes. Post-PCR Melting Curve Analysis would generate *T*_*m*_ values reflecting the sequence variations in the probe-binding region of the amplicons. Due to the possible existence of polymorphic SNPs sites in the probe-binding regions, a series of *T*_*m*_ rather than a single *T*_*m*_ for one allelic type would be generated. The probe was designed in such a way that it is complementary with none of the sequence variants at these polymorphic SNP sites. Consequently, the *T*_*m*_ values for one allelic type would be always lower than another allelic type. A series of binary codes could be obtained when multiple SNPs are genotyped. The concatenated serial binary codes are defined as MTs. The MTs can be further linked to STs or CCs.

### 2.4. MLST

MLST was performed on selected representative strains of major PFGE patterns as described previously. MLST was performed on all the strains by sequencing the internal fragments of seven housekeeping genes (arcC, aroE, glpF, gmK, pta, tpi, and yqiL). The loci were amplified using the primers and conditions recommended on http://www.mlst.com/ server. Sequencing was performed by BGI (Shenzhen, China). Sequences were analyzed online (http://pubmlst.org/vparahaemolyticus/) to assign allele numbers and define STs. The clonal complexes (CCs) of* S. aureus* were analyzed by goe-BURST [11] of Phyloviz software (http://www.phyloviz.net/) [12]. Those STs that share identical alleles at six of the seven MLST loci with at least one other ST were classified as one CC [13].

### 2.5. Susceptibility Testing

Susceptibility testing was performed on all* Staphylococcus aureus* strains using the Kirby-Bauer disc diffusion method in accordance with the performance standards for antimicrobial susceptibility testing recommended by the Clinical and Laboratory Standards Institute (CLSI) to 19 antimicrobial agents [14]. And we also used MIC method (Broth Dilution Method) to test* S. aureus* resistance to Vancomycin. Multidrug resistance was arbitrarily defined as resistance of* S. aureus* to three or more distinct antimicrobial classes. MCA were used to ensure methicillin-resistant* S. aureus* (MRSA, defined as* mecA*-positive strains). MRSA strains isolated in this study were included in the multidrug resistant (MDR) category irrespective of their susceptibility profiles. But we did not regard intermediate resistance as the antibiotic resistance. Results were analyzed using WHONET5.4 to determine resistance profiles (http://www.whonet.org.cn/index.html).

## 3. Data Analysis

Bacteriologic and patient data were compiled in an electronic database using Excel (Microsoft). Quantitative variables were summarized as mean ± SD and qualitative variables as proportions (%). In descriptive statistics, frequency and proportions were calculated for categorical variables. BioNumerics version 6.6.4 was used to cluster the outcomes of PFGE including picture processing and antibiotics susceptibility as well as isolated locations. As for MLMT and MLST analysis, Phyloviz software and Adobe Illustrator were used to deal with the calculation and picture adjustment together. As for antibiotic susceptibility, we use WHONET 5.4 to analyze the outcomes of 19 antibiotics.

## 4. Result

**Figure 1:**
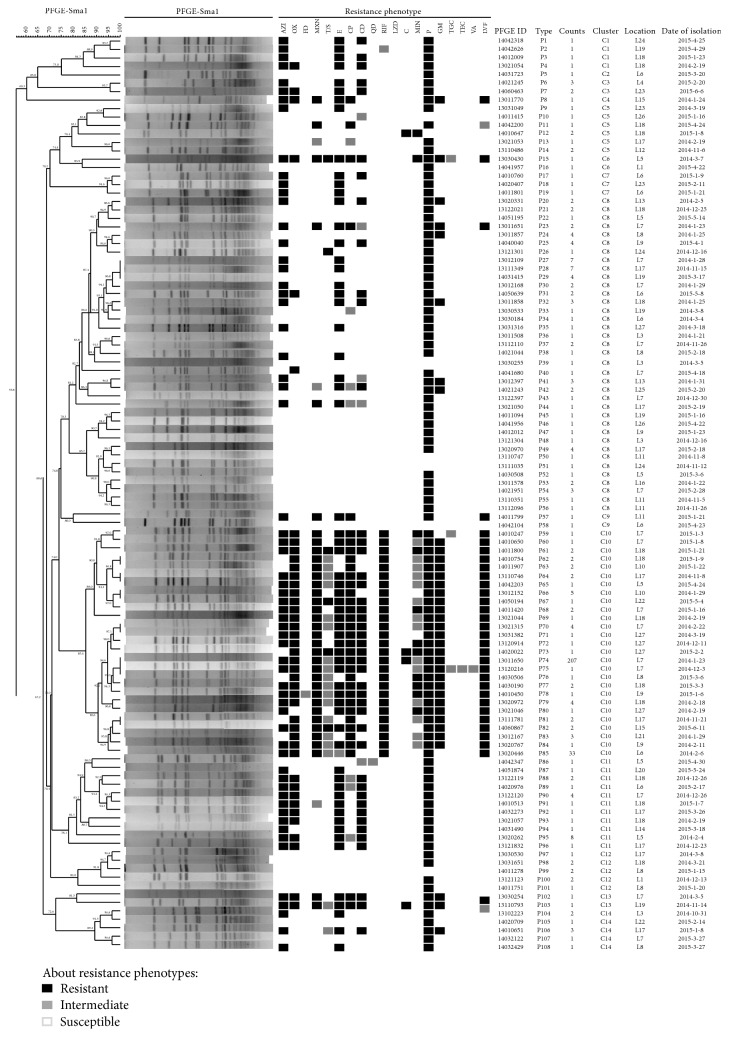
The PFGE cluster analysis of 108 representative* S. aureus* strains.

426 strains of* S. aureus* were conducted by PFGE using* S-maI* to cut into 15~20 pieces (20 kb~1200 kb). According to 100% similarity clustering standard, they were divided into 108 genotypes (P1~P108). MLMT resolved 426 strains into 20 MTs; among them, the most common MT was MT-1, which was composed of 284 strains and accounted for 66.67% of the total strains. And then, 108 representative strains were clustered into 14 clusters with 75% similarity standard (C1~C14). The similarity of all strains was from 55.8% to 100%, and discriminatory index (D) was 0.748 (Figure 1).

In Figure 1, L1~L27 represent different sources as follows orderly: L1 department of traumatic orthopedics, L2 department of ENT, L3 department of obstetrics and gynecology, L4 department of hepatobiliary surgery, L4 department of cadres health, L5 articular surgery, L6 respiratory medicine, L7 department of neurology, L8 ICU, L9 emergency department, L10 department of spine surgery, L11 department of geriatrics, L12 department of urinary surgery, L13 department of endocrinology department, L14 department of dermatology, L15 department of general surgery, L16 department of pediatrics, L17 department of medical oncology, L18 neurosurgery, L19 department of pediatrics, L20 department of anesthesia, L21 department of gastrointestinal surgery, L22 department of gastroenterology, L23 department of cardiology, L24 department of thoracic surgery, L25 department of vascular surgery, L26 department of hematology, L27 department of traditional Chinese medicine. Abbreviations are as follows: P: Penicillin; LVF: Levofloxacin; OX: Oxacillin; NXN: Norfloxacin; CP: Ciprofloxacin; GM: Gentamicin; RIF: Rifampicin; AZI: Azithromycin; E: Erythromycin; CD: Clindamycin; MIN: Minocycline; T/S: Trimethoprim and Sulphamethoxazole; C: Chloramphenicol; FD: Furantoin Tigecycline; Q/D: Quinupristin/Dalfopristin; TGC: Teicoplanin; LZD: Linezolid; VA: Vancomycin.

**Figure 2:**
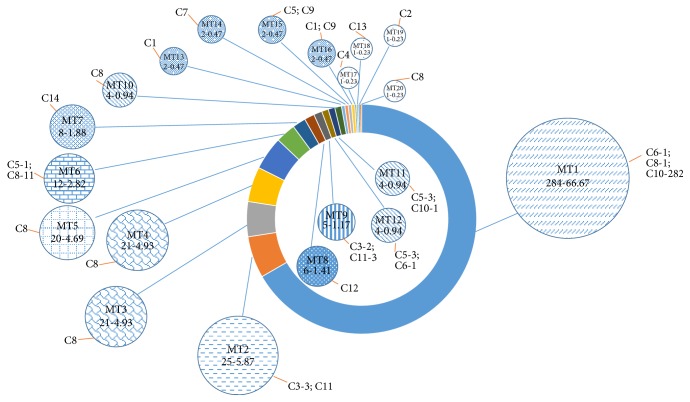
MLMT analysis results of 426* S. aureus* strains with PFGE. The number-frequency (%) of each MT is given together with the number of the corresponding PFGE clusters and the type and number of PFGE clusters of all the MTs obtained from the 426 strains. The size of the pies illustrates the relative number of MTs but not in a true scale.

As we can see, advantages of clusters are C10, 285/426 (66.90%); C8, 75/426 (17.61%); C11, 22/426 (5.16%), which were predominated and together accounted for 89.67% of the strains. The remaining 44 strains belonged to 11 sporadic profiles that were separated from 27 different sites. The clone strains distribute into concentrated sites, for instance, the largest number of P74 which belonged to C10 contains 206 strains (48.36%) from the neurology or neurosurgery wards, and most of the strains similarity may be up to 100%. Compared with the outcomes of PFGE, MLMT resolved 426 strains into 20 MTs (MT1~MT20); among them, MT-7 belonged to C14, MT-13 belonged to C1, MT-14 belonged to C7, MT-19 belonged to C2, MT-17 belonged to C4, and MT-3, MT-4, MT-5, MT-10, and MT-20 belonged to C8 (Figure 2). Association analysis between MT and clusters of PFGE for the 426 strains showed that MLMT results fully agreed with the theoretical predication.

**Figure 3:**
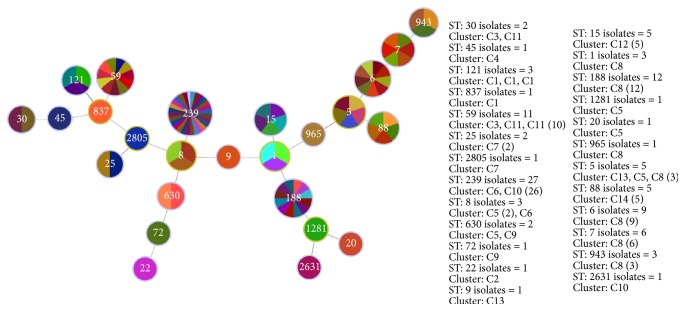
MST of 108 representative strains by MLST. MST: minimum spanning tree.

In order to further analyze the genotype P74 and other current strains, we used MLST as a combined method to compare with global prevalence clones. 108 representatives were typed by MLST (one randomly selected per PFGE type composed of more than one strain). MLST revealed a total of 25 different sequence types. ST239 (PFGE types = 27, containing C6, C10), ST188 (PFGE types = 12, containing C8), and ST59 (PFGE types = 11, containing C11) are accounted for most part of the representative strains (Figure 3).

Different MLST types shared common PFGE patterns (e.g., C3 and C22 both belonged to ST30). Vice versa, several strains with different MLST shared the same PFGE patterns (e.g., ST121 and ST837 included C1, Table 2).

As for antibiotic susceptibility, this study also tried to figure out the correlation between genetic background and antibiotic susceptibility. Figure 1 shows the antibiograms of the 108 representative* S. aureus* strains. Some clones, such as P6 or P7, showed high heterogeneity in their antibiograms, which belong to the same cluster patterns in the meantime. Of the 426* S. aureus* strains, 96.48% showed resistance to Penicillin, 77.70% to Levofloxacin, 70.89% to Oxacillin, 53.05% to Azithromycin, 68.54% to Norfloxacin, 67.84% to Ciprofloxacin, 64.32% to Gentamicin, 63.85% to Rifampicin, and 44.13% to Clindamycin (>50% would be recognized as resistant to some antibiotics in China). Ten strains (2.34%) were susceptible to all antibiotics (Table 3).

Multidrug resistance to one, four, five, six, seven, eight, nine, ten, eleven, and twelve antibiotics in addition to Oxacillin was observed for 0.7%, 5.87%, 0.94%, 12.21%, 15.26%, 5.40%, 17.37%, 9.39%, 3.05%, and 0.7% of the strains, respectively (Table 4). The most prominent combinations of resistance phenotypes are shown in Figure 1. And in Table 4, we can figure out that most of the MRSA strains isolated were resistant to at least four antimicrobial agents (including Oxacillin), emphasizing that antibiotic resistance remains a problem and underlines the importance of infection control measures.

## 5. Discussion

*Staphylococcus aureus* is among the most frequently identified antimicrobial drug-resistant pathogens worldwide and has evolved in a relatively few lineages. It has been demonstrated that some lineages are ecologically highly successful and that most strains belong to pandemic clones. Surprisingly, there are not many studies reporting data about all types of* S. aureus* infections in a general population of Xiamen City, China, in the last decade. This study provides the first comprehensive description about the epidemiology of* S. aureus* at a tertiary referral hospital in Xiamen.

The bacteriologic data is consistent with the research by Heipel et al. [15]; active surveillance by an infection control practitioner together with neurosurgeons may enhance significantly the sensitivity for detecting surgical site infection (SSI) after neurosurgery. Moreover, in a French national-based surveillance program, the SSI rate showed higher prevalence of* S. aureus*, and this confirms our study that great part of them were from surgical wards which seems to be a factor increasing the risk of* S. aureus* infection [16].

We characterized the* S. aureus* strains by using different molecular typing tools. After analysis of the data, we found interesting clinical and epidemiological findings. First, PFGE type 74 (C10; MT-1) was dominant among the 426 strains, a situation not described in other countries. In the meantime, we found a high degree of clonality of the strains obtained in this study, which demonstrates that most strains belong to pandemic clones. On the other hand, we found that PFGE, MLMT, and MLST presented a good correlation for most* S. aureus* strains.

The PFGE analysis revealed 108 different genotypes that included 3 predominant clones (C10, C8, and C11). Moreover, the P74 strains were all from neurology or neurosurgery wards and presented aggregation in the same isolation sites. Many studies have demonstrated that a clonal spreading can cause nosocomial outbreaks, so P74 is a great threat which would need more attention. Besides, PFGE is known to be a highly discriminatory and valuable technique for the typing of* S. aureus* [17] and has been used for staphylococci for local investigations and national surveillance in China. It has been argued that the stability of PFGE may be insufficient for its application to long-term epidemiological studies due to the high degrees of genetic variation that have been observed among pandemic clones with a long evolutionary history [18]. However, we have not found that the predominant clones in this study undergo significant changes during one year and half.

According to many reports, ST239, a typical Hungarian/Brazilian clone which belonged to clonal complex 8 (CC8), is the major endemic clone in many Asian countries, although recent studies show that it is being gradually replaced by other emerging clones as was also observed in our study (containing PFGE type 74, with the largest number). It was absent in other major global* S. aureus* strains from divergent clonal and geographical backgrounds. It is likely that the South American strains associated with ST239* S. aureus* are clustered within a uniform phylogenetic clade highly distinct compared to the Asian clade. This suggests that patients may represent an important reservoir for* S. aureus* dissemination within the hospital, when admitted as inpatients, and reinforces the observation of Alcoceba et al. [19].

Though several studies reported incidences of ST59 prevalence in Chicago of United States, there is a paucity of data on the molecular epidemiology of such clones. However, in our study, similar observations were found that ST59 may vary from other current clones from different locations. And we suspect that it might be community-acquired infection because ST59 carry Panton Valentine Leukocidin (PVL) which is associated with major CA-MRSA lineages such as ST1, ST30, ST8, and ST59 [20]. Few studies have documented the epidemiology of CA-MRSA as cause of health-care-onset infections in China and it should become another concerned problem in our hospital. On the other hand, PVL may be the unusual toxic gene both in community and in hospital.

In fact, the present work provides important epidemiological information on* S. aureus* in this tertiary referral hospital. More clinical and sequencing data should be obtained worldwide to help elucidate the global epidemiology and evolution. To confirm the sources and affinity of these strains mentioned above, we used three molecular typing methods (PFGE, MLMT, and MLST) to explore the clonal spread of* S. aureus* between different hospital wards. And the results of this study conclude that the combination of PFGE and MLST is more discriminatory as compared to using a single method only and will allow us to better monitor and recognize changes in* S. aureus* epidemiology over time.

In Europe and the United States, certain clinical strains of* S. aureus* are sensitive to antibiotics which are commonly used in clinic except for Penicillin. So the condition of drug resistance has not attracted enough attention [21, 22]. But in our country, multidrug resistance of* S. aureus* is highly reported in recent years, especially Erythromycin, Clindamycin, Oxacillin, and Penicillin [23]. The present study revealed that 70.19% of the strains were multiresistant and that 51.24% were resistant to at least 7 antimicrobial agents. Some MDR bacteria have a common drug resistance spectrum (Levofloxacin, Oxacillin, Norfloxacin, Ciprofloxacin, Gentamicin, Rifampicin, and Azithromycin). As we know, in these years, the emergence of VISA and hVISA was reported abroad. Although we did not find any strain resistant to Vancomycin until now, it still should be a caution for us to use Vancomycin to cure* S. aureus* infection in patients. From another perspective, the strains identified to the same cluster seem to have similar resistance phenotypes which enhance the evidence of the genotyping.

However, the current retrospective study lacks an analysis of hospital environmental specimen and may become the limitation of our further research. But the affinity analysis of* S. aureus* provides important information on the hospital epidemiology and infection control surveillance and monitoring. Even for other hospitals in Xiamen, this study is also important to demonstrate the long-term persistence of an endemic clone of* S. aureus* responsible for nosocomial infections within a hospital.* S. aureus* has been recognized as a major pathogenic species in the hospitalized patients which suggested that further investigations should be performed to elucidate its mode of transmission and the* S. aureus* reservoir and to identify specific preventive measures that could minimize the emergence and spread of such bacteria in the hospital environment, especially in the neurology and ICU.

In summary, we have demonstrated that (I) several of the lineages that are predominant in the world are present in our hospital, (II) ST188 may be the new prevalence strains in this study without reports in other former studies, (III) the combination of PFGE, MLMT, and MLST is more discriminatory as compared to using a single method only and will allow us to better monitor and recognize changes in* Staphylococcus aureus* epidemiology over time, (IV) clonal complex predictions based on PFGE and MLMT could be confirmed by MLST, and (V) there is still a serious problem about MDR strains.

## Figures and Tables

**Table 1:** Demographics and sources of 426 *S. aureus *strains.

Characteristics	Demographic outcomes	
	*N*	*N*%
*Sex*		
Male	243	57.04%
Female	183	42.96%
*Age (years) mean ± SD*	65.96 ± 17.86	
<30	13	3.05%
30~	54	15.73%
45~	76	17.84%
60~	105	24.65%
75	178	41.78%
*Patient location*		
Medical ward	159	37.32%
Surgical ward	199	46.71%
ICU	68	15.96%
*Duration of hospital stay (days) before culture*		
0~9	251	58.9%
10~19	83	19.48%
>20	92	21.60%
*Sources of specimen*		
Blood	13	3.05%
Bile	1	0.23%
Secreta	35	8.22%
Fester	6	1.41%
Sputamentum	344	80.75%
Pleural effusion	1	0.23%
Synovial fluid	1	0.23%
Drainage liquid	7	1.64%
Throat swab	8	1.88%
Midstream urine	4	0.94%
Others	6	3.05%

*Note*. Values are mean ± SD of age.

**Table 2:** MLST, MLMT, and PFGE cluster analysis of 108 *S. aureus* strains.

MLST	MLMT	PFGE clusters	PFGE types	Counts
ST1	MT-10	C8	P44, 39, 31	3
ST5	MT-6	C5, C8, C13	P12; P23, 41, 43; P102	5
ST6	MT-1, MT3	C8	P36, 27, 30, 33, 35, 28, 37, 38, 29	9
ST7	MT-4	C8	P32, 20, 34, 26, 25, 40	6
ST8	MT-12	C5, C6	P9, 10; P16	3
ST9	MT-18	C13	P16	1
ST15	MT-8, MT-12	C12	P97, 98, 99, 100, 101	5
ST20	MT-11	C5	P13	1
ST22	MT-19	C2	P5	1
ST25	MT-14	C7	P17, 19	2
ST30	MT-9	C3, C11	P6; P87	2
ST45	MT-17	C4	P8	1
ST59	MT-2	C11	P95, 93, 96, 88, 90, 91, 89, 94, 92, 86, 87	11
ST72	MT-16	C9	P58	1
ST88	MT-7	C14	P104, 105, 106, 107, 108	5
ST121	MT-13	C1	P1, 2, 4	3
ST188	MT-5	C8	P45, 46, 47, 48, 49, 50, 51, 52, 53, 54, 55, 56	12
ST239	MT-1	C6, C10	P15; P59, 60, 61, 62, 63, 64, 65, 66, 67, 68, 69, 70, 71, 72, 74, 75, 77, 78, 79, 80, 81, 82, 83, 84, 85, 86	27
ST630	MT-15	C5; C9	P11; P57	2
ST837	MT-13	C1	P3	1
ST943	MT-4	C8	P21, 22, 24	3
ST965	MT-20	C8	P42	1
ST1281	MT-11	C5	P14	1
ST2631	MT-11	C10	P76	1
ST2805	MT-14	C7	P18	1

**Table 3:** Antibiotic susceptibility of 426 *S. aureus* strains (19 antibiotic agents).

Antibiotics	Resistant	Intermediate	Susceptible	Resistance rate
Penicillin	410	0	15	96.24%
Levofloxacin	330	1	95	77.46%
Oxacillin	301	0	124	70.66%
Norfloxacin	292	3	130	68.54%
Ciprofloxacin	288	10	127	67.61%
Gentamicin	273	5	147	64.08%
Rifampicin	271	1	153	63.62%
Azithromycin	225	1	199	52.82%
Erythromycin	201	5	199	47.18%
Clindamycin	187	6	232	43.90%
Minocycline	105	136	184	24.65%
Trimethoprim and Sulphamethoxazole	46	207	173	10.80%
Chloramphenicol	28	1	396	6.57%
Furantoin	9	6	410	2.11%
Tigecycline	3	11	411	0.70%
Quinupristin/Dalfopristin	2	1	422	0.47%
Teicoplanin	2	0	423	0.47%
Linezolid	1	0	424	0.23%
Vancomycin	0	0	425	-

**Table 4:** Multiresistance phenotypes of 426 *S. aureus *strains resistant toother 18 antibiotics including Oxacillin.

Resistance profile	Number (%) of strains
Oxacillin + 1 antibiotic	3 (0.7%)
Oxacillin + 4 antibiotics	25 (5.87%)
Oxacillin + 5 antibiotics	4 (0.94%)
Oxacillin + 6 antibiotics	52 (12.21%)
Oxacillin + 7 antibiotics	65 (15.26%)
Oxacillin + 8 antibiotics	23 (5.40%)
Oxacillin + 9 antibiotics	74 (17.37%)
Oxacillin + 10 antibiotics	40 (9.39%)
Oxacillin + 11 antibiotics	13 (3.05%)
Oxacillin + 12 antibiotics	3 (0.70%)
